# Congenital deafness and vestibular disorders: a systematic literature review

**DOI:** 10.3389/fneur.2024.1463234

**Published:** 2024-09-17

**Authors:** Elisabetta Genovese, Erika Segato, Carlotta Liberale, Erica Zampieri, Daniele Monzani, Enrico Apa, Silvia Palma, Riccardo Nocini

**Affiliations:** ^1^Unit of Audiology, Department of Diagnostic, Clinical, and Public Health, University of Modena and Reggio Emilia, Modena, Italy; ^2^Unit of Otorhinolaryngology, Department of Head and Neck, University of Verona, Verona, Italy; ^3^Unit of Audiology, Department of Specialist Surgical Sciences, Fondazione IRCCS Ca’ Granda Ospedale Maggiore Policlinico, Milan, Italy; ^4^Unit of Audiology, Primary Care Unit, Modena, Italy

**Keywords:** congenital deafness, vestibular tests, deafness, vertigo, hypoacusia

## Abstract

**Introduction:**

Congenital deafness is a pathological entity that represents an economical and social burden, affecting up to 0.2% of newborns in Europe. Sensorineural hearing loss (SHL) is caused by a variety of factors, including congenital abnormalities, perinatal infectious diseases and genetic syndromes. The inner ear’s vestibular system, nestled alongside the auditory organs, is crucial for balance maintenance. Its close connection with the auditory system means that disturbances in one often coincide with disturbances in the other, highlighting their intertwined functions. With this review we aim to describe objective vestibular tests found in literature and to study their use for diagnosis of vestibular disturbances in patients affected by congenital deafness.

**Methods:**

The review is conducted with the Preferred Reporting Items for Systematic Reviews and Meta-Analysis (PRISMA) 2020 guidelines. The search string used was: *[(congenital deafness) OR (congenital hearing loss) OR (congenital hypoacusia)] AND [(vestibular disorders) OR (vertigo)]*. An initial abstract reading selection was made, and a subsequent comprehensive full-text reading. For each article, we identified the type of vestibular test utilized and its corresponding outcome.

**Results:**

Out of the initial—papers identified through the search string—articles met the eligibility criteria for further analysis through abstract and full-text reading. After further selection—articles were chosen for detailed examination, focusing on the data of patients.

**Conclusion:**

Congenital hearing loss profoundly affects a child’s development, especially in language and communication skills, and it is frequently associated with a pathological vestibular system. Early identification allows timely intervention with personalized therapies. In current literature, there is still no gold standard test to identify balance disorders in patients with congenital hearing loss. There is considerable variability on the subject due to the inclusion of diverse patients with various diagnoses, alongside a wide range of available technologies. Managing such conditions necessitates collaboration among healthcare providers, ensuring comprehensive care through prompt diagnosis and personalized treatment plans. Ongoing research aims to further improve screening methods and develop precision medicine approaches tailored to individual needs.

## Introduction

Congenital hearing loss is one of the most common forms of congenital disability in newborns worldwide and can affect one ear (unilateral hearing loss) or both ears (bilateral hearing loss). Despite the progress of neonatology, its incidence has remained constant in time. Hearing loss affects between 1 and 2 per 1,000 infants in the United States and in some categories of infants, such as those with familiarity for congenital deafness, the prevalence can be 10–20 times higher (1).

Screening programs have been implemented to allow timely treatment and avoid adverse consequences on the development of the child’s language and cognitive skills. About 50% of cases of congenital deafness are genetic, inherited by one or both parents. Genetic hearing loss can have both syndromic and non-syndromic causes. The other 50% are non-genetic (or environmental).

According to the site of lesion, hearing loss can be categorized in conductive (outer or middle ear) and sensorineural (inner ear or auditory nerve or central auditory pathway). Mixed hearing loss is defined as the cohexistence of the two (2).

Examples of congenital conductive hearing loss are external ear malformations, for example microtia, stenosis or atresia of the external auditory canal, that can occur both as a part of a clinical syndrome (i.e., CHARGE syndrome) or isolatedly, but also malformations of the ossicles, as ossicular chain fixation, usually part of a syndrome (e.g., Treacher-Collins syndrome, branchio-oto-renal syndrome, DiGeorge syndrome, Beckwith-Wiedemann syndrome).

Sensorineural congenital genetic hearing loss can be associated with syndromes as Waardenburg syndrome, Usher syndrome, Pendred syndrome, Alport syndrome, and Jervell and Lange-Nielsen syndrome or can be linked to isolated gene mutations, commonly *GJB2* and *STRC genes*.

Non-genetic causes of sensorineural congenital hearing loss are (3).

- *in-utero* infections by TORCH complex pathogens. The most common cause is congenital CMV, which can determine delayed or progressive sensorineural hearing loss (SNHL), both monolaterally and bilaterally. Also toxoplasma, rubella, Zika virus and syphilis can be responsible.
- Inner ear malformations, for example Enlarged Vestibular Aqueduct (EVA) Syndrome, manifesting with varying degrees of progressive deafness, sometimes associated with vestibular disorders
- Perilymph fistula, very uncommon, determining fluctuating severe SNHL, disequilibrium, and aural fullness

The vestibular system is constituted by peripheral structures linked to a complex central neural network, and contributes to give to the brain inputs regarding movement and orientation of the body in space.

Specifically, informations from the peripheral vestibular apparatus, located in the inner ear, are carried to the brainstem through the inferior and superior vestibular nerves (cranial nerve VIII). Those informations are represented by angular acceleration of the head in the space, detected by the sensory hair cells present in the ampullae of the semicircular canals, and linear acceleration, to which the maculae of the otolith organs, utricle and saccule, are dedicated. In the brainstem the vestibular nuclei receive peripheral vestibular inputs and integrate them with sensory afferences from visual and proprioceptive systems. Ultimately, motor effereces are conveyed to the brain and spinal cord, for the control of eye movement (vestibulo-ocular reflexes), balance maintenance and postural adjustments (vestibulospinal reflexes).

A dysfunction of this innately complex system can be peripheral or central and May present acutely or chronically.

A peripheral vestibular dysfunction is a pathology of the vestibular system itself: the membranous labyrinth and the superior and inferior vestibular nerves. On the other hand, a central vestibular disorder affects the central nervous system itself, for instance in trauma, stroke and demyelinating conditions (4, 5).

Similarly to congenital hearing loss, congenital vestibular disorders May be determined by genetic disorders, syndromic or isolated, but also be associated with structural anomalies, malformations and exposure to prenatal insults, either infectious noxae or ototoxic drugs.

The diagnosis of vestibular disorders May involve imaging, such as magnetic resonance imaging (MRI) when a central lesion is suspected from history of physical examination, or computed tomography (CT), but also clinical tests, with various degrees of complexity and availability, depending on the specific setting (4).

For example, in an emergency room setting, the HINTS examination (head impulse, nystagmus, test of skew) is a valid tool to differentiate central or peripheral causes of acute vertigo (6).

On the other hand, the diagnosis of vestibular dysfunctions May often be a challenge, because of symptom variability and patients’ comorbidities, requiring objective vestibular tests in order to reach an accurate diagnosis. These tests can identify the site of lesion (central vs. peripheral, side but also the specific structure involved, e.g., superior vs. inferior vestibular nerve), quantify vestibular function, assess compensation status and monitor disease’s progression (7).

Even though vestibular tests can give pathophysiological information about vestibular function, they are unable to determine a specific diagnosis, or the impact of a certain deficit on level of disability or quality of life.

Analysis and interpretation of eye movements represents one of the main tools to verify vestibular system function through its interaction with the visual system.

Pathologic nystagmus (spontaneous, gaze, positional, and positioning) can represent a sign of lesion of peripheral vestibular system as well as lesions of cerebellum or any site involved in the central vestibular pathway.

Videonystagmography (VNG) or electronystagmography (ENG) can be used to record nystagmus (whether spontaneous or induced by various stimuli, such as rotational, caloric, optokinetic, and gaze testing) and other eye movements, including saccades or pursuit movements during oculomotor assessments.

Vestibular evoked myogenic potentials (VEMPs) represent a neurophysiologic test to assess the function of utricule and saccule, the otholitic organs. In particular, the oVEMPs (ocular VEMPs) determine the function of the utricule and superior vestibular nerve while the cVEMPs (cervical VEMPs) evaluate the saccule and the inferior vestibular nerve.

Auditory pathway function (from CN VIII to the mesencephalon) can be evaluated through Auditory brainstem response (ABR), or Brainstem auditory evoked potentials (BAEP) (4, 7).

The association between SNHL and vestibular disorders is described in literature, however frequently underestimated by most professionals. Vestibular impairment has repercussions on gross motor functions, with delayed motor development and postural control, hindering the achievement of motor milestones like head control, independent sitting and walking (8). Fine motor functions are however usually preserved, in the absence of CNS involvement (9).

The prevalence of vestibular and balance disorders in children with SNHL is elevated, in fact some estimates indicate that almost 70% of children with SNHL have vestibular system impairment, with 20–40% having severe bilateral vestibular loss (8). Eventually, the integration between pyramidal and extrapiramidal motor systems and visual and somatosensory systems, associated with intellectual development, compensate for vestibular failure, and these children catch up with their peers in terms of development and motor function (9).

The aim of this review is to analyze the role of objective vestibular tests in studying vestibular function in patients with congenital deafness.

## Materials and methods

**Figure 1:**
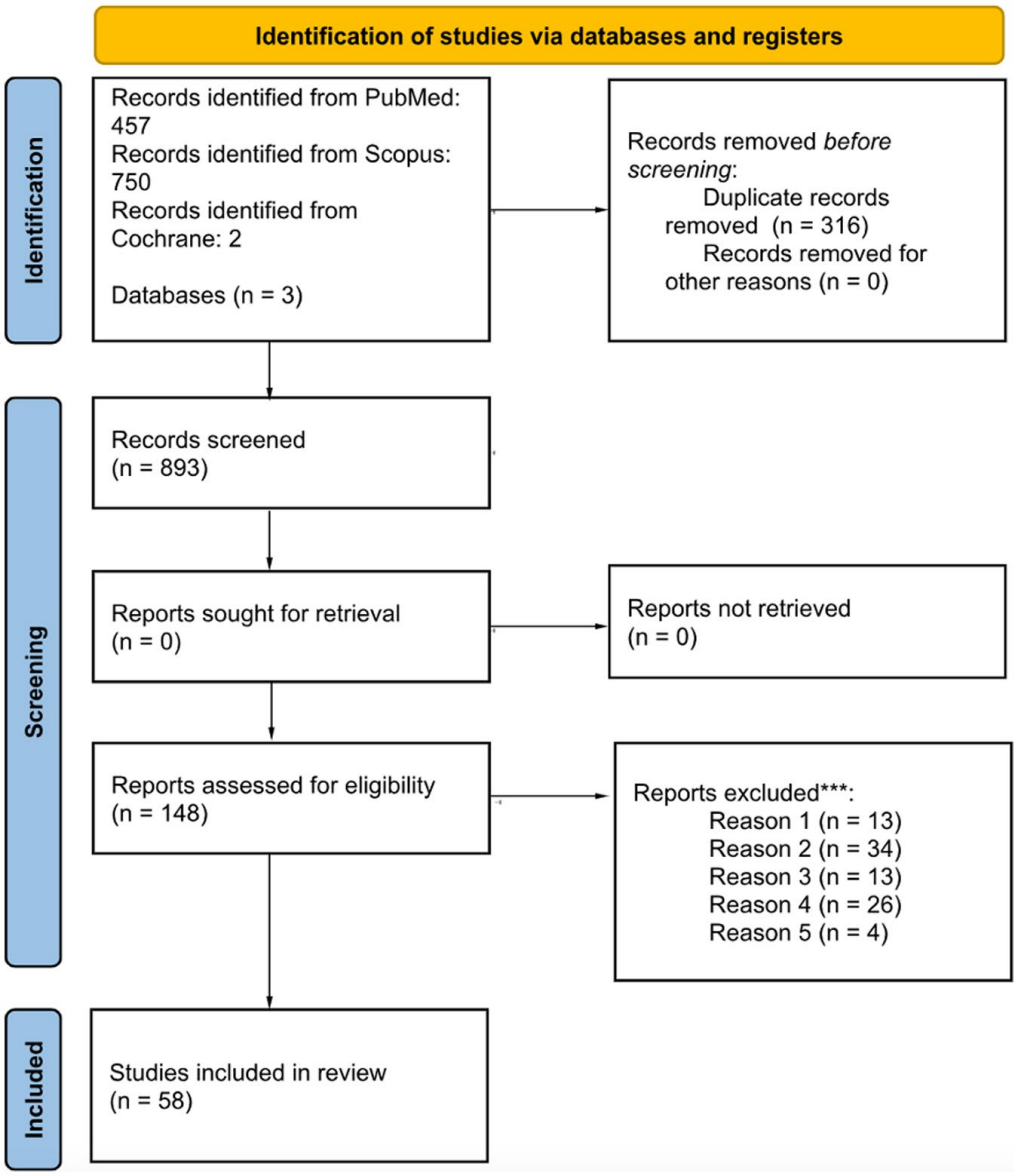
Literature selection following the PRISMA statement guidelines. *Consider, if feasible to do so, reporting the number of records identified from each database or register searched (rather than the total number across all databases/registers). **If automation tools were used, indicate how many records were excluded by a human and how many were excluded by automation tools. ***Reason 1 = absence of a diagnosis of congenital deafness; reason 2 = lack of objective vestibular tests; reason 3 = unavailability of full-text articles; reason 4 = insufficient data; reason 5 = cochlear implant.

This review was conducted with the preferred reporting items for systematic reviews and meta-analysis (PRISMA) 2020 guidelines (See Figure 1). The research was carried out using the Pubmed, Scopus and Cochrane database with the following research string: *[(congenital deafness) OR (congenital hearing loss) OR (congenital hypoacusia)] AND [(vestibular disorders) OR (vertigo)]*. Three reviewers (ES, CL, and EZ) performed the literature search and the abstract and full text reading. All the articles found were included, without any period restriction. Last research on the database was performed in January 2024. The inclusion criteria for the initial abstract reading selection were as follows: articles focusing on congenital hearing loss, inclusion of objective vestibular tests, relevance to the inherent topic, and written in English. The exclusion criteria for this phase included the absence of evidence for congenital hearing loss, lack of objective vestibular tests, unrelated topic, and non-English papers. Following the initial abstract-based selection, a comprehensive full-text reading was conducted, incorporating additional criteria.

The inclusion criteria for the second selection phase were: individuals with congenital deafness who had undergone objective vestibular tests, the availability of full-text articles, and complete data. Exclusion criteria included the absence of a diagnosis of congenital deafness, lack of objective vestibular tests, unavailability of full-text articles, insufficient data (i.e., lack of specific data regarding the number of patients and the type of deafness) and the presence of cochlear implant. The selected articles were then analyzed in detail, extracting data about: type of study design, number of patients, sex and age of patients, diagnosis and type of deafness, types of vestibular disorders and their related symptoms, audiometry, and vestibular tests.

## Results

A total of 1,209 papers were selected using the aforementioned research string. A total of 316 duplicated articles were eliminated resulting in 893 articles eligible for further analysis. First, an abstract reading selection was made according to the inclusion criteria and 148 abstracts were selected. After this, a further exclusion reading full-length papers was made, with 59 articles selected, following the pre-established criteria. The process of literature selection following the PRISMA statement guidelines is reported in Figure 1.

A total of 1,700 patients (sex was not always available) were reported in the selected papers. The articles analyzed cover a time span from 1960 to 2023. They examine patients with congenital sensorissneural hearing loss, both unilateral and bilateral. The main vestibular symptoms considered by each article were analyzed, including vertigo, ataxia, dizziness, nausea, balance problems, delayed walking, and paroxysmal positional vertigo. Furthermore, the various types of vestibular tests used and their results were analyzed in detail. The details of patients’ results are reported in Table 1.

**Table 1:** Details of patients’ results.

Author	Year	*N*	Sex	Age	Diagnosis	Type of deafness	Vestibular disorders and symptoms	Vestibular tests	Results of vestibular tests
Everberg (21)	1960	122	76 M 46 F	7–21 y (range)	–	USNHL	–	sNy, rotatory and caloric tests	Vestibular function was absent on the deaf side in 28%
Hageman et al. (22)	1977	12	5 M 7 F	24.3 y (mean)	Waardenburg Syndrome	6 USNHL 4 BSNHL	–	sNy and caloric tests	40% pNy, 50% alterations in caloric test
Karmody et al. (23)	1982	4	M	15.25 y (mean)	–	BSNHL	Episodic vertigo (endolymphatic hydrops)	Caloric tests with ENG	-
Nishioka (24)	1982	2	1 M 1 F	6–9 y (range)	Complex malformation of the inner ear	USNHL	–	Caloric tests	No response to caloric tests
Nishida et al. (25)	1983	72	41 M 31 F	12 y (mean)	Congenital rubella syndrome	SNHL	–	Caloric tests, righting reflex test	24 (30%) had vestibular hypofunction
Elverland et al. (26)	1983	1	M	–	Oval window fistula	USNHL		Caloric tests	No response to caloric tests in the affected ear
Kumar et al. (27)	1984	70	–	–	Usher Syndrome	BSNHL	2/ 70 patients with ataxia	sNy and induced nystagmus and caloric tests	vestibular function was decreased in all 24 patients with profound hearing loss
Schweitzer et al. (28)	1984	1	F	21	Waardenburg Syndrome	BSNHL	DIZZINESS, nausea, difficult spatial orientation at night	Monocular ENG recordings; ENG for OKN test; caloric tests with ENG	–
Strauss (29)	1985	3	–	5.5 y (mean)	CMV	Congenital HL	–	Caloric tests	–
Enbom et al. (30)	1991	6	–	13.8 y (mean)	Usher Syndrome	BSNHL	–	Rotary chair testing	–
Hill et al. (31)	1992	2	1 M 1 F	42 y (mean)	Kallmann Syndrome	BSNHL	Clumsy child in F	caloric test, rotary chair testing, sway tests	negative
Wagenaar et al. (32)	1995	17	9 M 8 F	55.6 y (mean)	Usher Syndrome type 1	BSNHL	None	ENG	negative
van Aarem et al. (33)	1995	10	3 M 7 F	48.8 y (mean)	Usher syndrome type 2A	BSNHL	None	ENG saccades, smooth pursuit, and OKN, responses; caloric tests, rotary chair testing	Some vestibular abnormalities
Rosenberg et al. (34)	1996	5	2 M 3 F	56 y (mean)	Retinitis pigmentosa	Congenital HL	Balance problems	caloric tests (only in 1 case)	–
Braverman et al. (35)	1996	15	6 M 9 F	31.6 (mean)	Mitochondrial DNA mutation	SNHL	–	ENG, saccades, smooth pursuit, and OKN responses; caloric tests	1 patient with decreased caloric response bilaterally; 1 patient with unilateral canal paresis
Selz et al. (36)	1996	5	3 M 2 F	8–17 y (range)	Congenital deafness	SNHL	–	ENG, rotary chair testing	abnormally increased amounts of nystagmus
Pfister et al. (37)	1999	14	–	–	Duchenne muscular dystrophy	SNHL	–	ENG, caloric tests	–
Guyot et al. (38)	1999	1	M	10 y	CHARGE Syndrome	BMHL	Delay in the development of walking	ENG, caloric tests	complete absence of nystagmic response to bithermal caloric
Ozeki et al. (39)	1999	1	M	31 y	–	BSNHL	–	Caloric tests, VEMPS	normal
Luxon et al. (40)	2003	22	10 M 12 F	12–47 y (range)	Pendred syndrome	BSNHL	–	ENG, caloric tests	Unilateral or bilateral abnormality
Sheykholeslami et al. (41)	2004	3	3 F	15,3 y (mean)	Large vestibular aqueduct	BSNHL	Giddiness and dizziness, episodes of vertigo and nausea.	Caloric tests, VEMPS	VEMPs were asymmetrical
Mäki-Torkko et al. (42)	2005	3	1 M 2 F	17 m (mean)	–	BSNHL	Difficult walking	HIT, rotary chair testing	Both tests pathological
Dikkers et al. (43)	2005	3	2 M 1 F	–	–	USNHL	None	Caloric test	Caloric inexcitability
Weiss et al. (44)	2006	1	M	2 y	Usher syndrome type 1	SNHL	Imbalance, delayed motor development, and cyclic vomiting	Rotary chair testing, ENG, computerized platform posturography	Severe bilateral vestibular hypofunction
Ebermann et al. (45)	2007	8	6 M 2 F	–	Mutation of the DFNB59 Gene	SNHL	–	Caloric tests, computerized platform posturography	Abnormal computerized platform posturography
Zhou et al. (46)	2008	54	22 M 32 F	7 y (mean)	EVA syndrome	SNHL or MHL	–	VEMPs	VEMPs with abnormally low thresholds and higher amplitude
Kaga et al. (9)	2008	20	11 M 9 F	31–97 m (range)	–	BSNHL	–	Caloric tests, rotary chair testing, VEMPs	Abnormal results
Schraders et al. (47)	2010	38	–	–	DFNB25 Mutations of GRXCR1	SNHL	–	Rotary chair testing	–
Kasai et al. (48)	2010	21	17 M 4 F	–	GJB2 related deafness	SNHL	–	VEMPs, caloric tests	Abnormal results
Jafari et al. (49)	2011	30	16 M 14 F	6–9,4 y (range)	–	SNHL	–	ASNR, VEMPs	Abnormal results
Song et al. (50)	2012	5	1 M 4 F	17.8 y (mean)	EVA syndrome	SNHL	BPPV	sNy, caloric tests, VEMPs	-
Kulkarni et al. (51)	2012	3	M	–	Jervell and Lange-Nielsen syndrome	BSNHL	Delayed motor development	Halmagyi head thrust test standing on foam, eyes closed tandem gait impulsive rotation, caloric tests	Abnormal results
van Beelen et al. (52)	2014	5	2 M 3 F	24,4 y (mean)	Barakat syndrome	BSNHL	Dizziness and instability	Rotary chair testing, caloric tests, ENG	–
Zhou, G et al. (53)	2014	278	119 M 159 F	10.5 (mean)	–	CHL SNHL	–	cVEMPs	Abnormal cVEMPs in 25% of children with complaints of dizziness or vertigo
Van Houtte et al. (54)	2014	2	1 M 1F	unknown	Valproic acid during pregnancy	Mild unilateral CHL	Global motor impairment	Caloric tests, cVEMP, Movement Assessment Battery, Bruininks Ozeretsky test-2	F: mild isolated directional preponderance of 26% to the right
White et al. (55)	2015	4	3 M 1 F	9–54 (range)	Enlarged Aqueduct Syndrome	Profound SNHL	Variable: dizziness, episodic vertigo, acute vertigo attack, disequilibrium	Rotary chair testing, caloric tests, positioning testing	
Bernard et al. (56)	2015	48	–	34.7 months (mean)	Congenital CMV	BSNHL	Deficit of both canal and otolith function	Caloric tests; EVAR; HIT; OVAR; cVEMPs	90.4% of the children had canal dysfunction, and 86.5% (45/52) had otolithic dysfunction.
Lin et al. (57)	2016	30	8 M 22 F	Group A 40 + −14; group B: 34 + −12	–	CHL	Rotatory vertigo	oVEMP, cVEMP and caloric tests	Higher percentages of absent oVEMPs and caloric areflexia in congenitally deaf patients
Kovač et al. (47)	2017	2	1 M 1 F	–	Loss-of-function ILDR1 mutation	Profound progressive SSNHL	None	Caloric tests	No alterations
Kletke et al. (58)	2017	33	21 M 12 F	10.24 ± 5.43 y (mean)	Congenital SNHL and vestibular impairment	SNHL	Vestibular dysfunction and functional impairments in static and dynamic balance	Caloric tests, rotary chair testing and/or vHIT, cVEMPs and oVEMPs	Patients with combined congenital SNHL and vestibular dysfunction had an 80% chance of having an ocular abnormality
Magliulo et al. (59)	2017	7	3 M 4 F	32.4 y (mean)	Usher’s Syndrome	CHL	Sporadic dizziness	Caloric tests, CVEMPs, OVEMPs, and vHIT	80% showed pathological oVEMPs. 40% reported bilateral absent or abnormal values of cVEMPs.
JanssensdeVarebeke et al. (60)	2018	2	M	9 y (mean)	COCH gene variation	BSHL	–	VNG, caloric tests	Hypo-reflective caloric response
Wesdorp et al. (61)	2018	14	-	-	DFNB77	BSHL	None	cVEMPs, vHIT and ENG	There was no evidence for involvement of the vestibular system
Kimura et al. (62)	2018	195	–	3 m–6 y (range)	Inner ear malformations	Profound HL	–	Rotary chair testing	84.1% normal response; 5.6% poor response, 10.3% no response
Dhondt et al. (63)	2019	5	3 M 2 F	2–7 y (range)	Congenital CMV	SHL	Vertigo, nausea, vomiting, hypotonia, instability, headache, photophobia, nystagmus	vHIT, rotary chair testing, caloric tests, cVEMPs, oVEMPs	4/5 were diagnosed with a peripheral vestibular deficit.
Takeuti et al. (64)	2019	31	16 M 15 F	23.9 +/− 7.3 y (mean)	Idiopathic etiology *n* = 6 Genetics *n* = 7 Congenital rubella *n* = 7 Congenital syphilis *n* = 1 Ototoxicity *n* = 2 Prematurity *n* = 1 Meningitis *n* = 2 Unknown *n* = 5	SHL	–	cVEMPs	Higher propensity of presenting altered cVEMPs results
Kotait et al. (65)	2019	32	18 M 14 F	11.50 ± 2.33 y (mean)	Non-syndromic hearing loss *n* = 25, Warden-burge’s syndrome *n* = 1	SHL	–	cVEMPs, oVEMPs; rotary chair testing, Sinusoidal harmonic acceleration test	Delayed latencies and reduced amplitudes in both oVEMPs and cVEMPs
Dehadaray et al. (66)	2020	80	44 M 36 F	6 m to 40 y (range)	–	Congenital HL	–	Caloric tests; rotary chair testing	–
Lazar et al. (67)	2021	10	8 M 2 F	–	Congenital CMV	BSNHL/USNHL	Delayed motor development	VHIT and VEMPs	Six bilateral dysfunction and one unilateral
Wolter et al. (19)	2021	18	9 M 9 F	14.28 y (mean)	Usher syndrome (7)/Meningitis (4)/Cochleovestibular anomaly (3)/Unknown etiology (3)/CMV (1)	BSNHL/USNHL	Balance impairment	Caloric tests, rotary chair testing, vHIT and cVEMPs	Absent calorics bilaterally and reduced VOR gain on rotational chair testing or vHIT and absence of VEMPs response
D’Esposito et al. (68)	2021	1	F	26 y	Usher syndrome	BSNHL	None	vHIT and caloric tests.	Normal
Wang et al. (69)	2021	44	23 M 21 F	2.8 ± 3.8 y (mean)	Various genetic disorders syndromic and non-syndromic	BSNHL	Delayed motor development	VNG during rotary chair testing, vHIT, and/or cVEMPs, oVEMPs	–
Pinninti et al. (70)	2021	7	–	–	Congenital CMV	BSNHL (1)/USNHL (6)	–	vHIT, rotatory chair test, cVEMPs, cDVA	Abnormal VOR *n* = 4; abnormal cVEMPs *n* = 3
Kimura et al. (71)	2022	15	5 M 10 F	39 m (mean)	Semicircular canal aplasia and hypoplasia (aplasia → CHARGE syndrome; ipoplasia → CHD7 mutations)	Congenital hearing loss	Delayed gross motor development	Damped rotational chair test examining horizontal VOR	Severe dysfunction of VOR
Martens et al. (72)	2022	254	125 M 129 F	7.4 +/− 2.4 m (mean)	Various genetic disorders syndromic and non-syndromic	BSNHL/USNHL	–	cVEMPs	Abnormal results were found in 13.8% of the infants (35 of 254).
Grijpink et al. (73)	2023	1	M	13 y	Congenital CMV	SNHL	Recurrent episodes of tinnitus, vertigo and nausea.	vHIT, rotary chair testing, caloric tests, cVEMPs	Rotary chair testing bilateral hypofunction, caloric testing bilateral areflexia and cVEMPs bilaterally absent
Velde et al. (74)	2023	4	–	–	Autosomal dominant non-syndromic hearing loss WFS1	SNHL	–	Smooth pursuit, gaze, OKN, fixation suppression, and saccade tests, caloric tests and rotary chair testing, vHIT, oVEMPs and cVEMPs	Caloric tests and vHIT were within the normal range in all subjects
Kokkola et al. (75)	2023	2	–	–	Congenital CMV	USNHL	Bilateral vestibular disfunction	vHIT	Abnormal gain
Dasgupta et al. (76)	2023	2	M	16,5 y (mean)	X-Linked Gusher Disease DFNX2	Severe mixed hearing loss	Delayed motor development; unsteadiness; postural instability; difficult ambulation in darkness, in reading, in playground activities.	VNG with and without optic fixation, VST, vHIT, SHIMP, cVEMP, SVV	Patient 1: absent cVEMP on the left, abnormal VST but normal SHIMP, SVV and VNG; patient 2: abnormal cVEMP, with abnormal VST but normal SHIMP, SVV and VNG.

USNHL, unilateral sensorineural hearing loss; BSNHL, bilateral sensorineural hearing loss; sNy, spontaneous nystagmus; pNy, positional nystagmus; ENG, electronystagmography; OKN, optokinetic nystagmus; VEMPs, vestibular-evoked myogenic potentials; cVEMPs, cervical vestibular-evoked myogenic potentials; oVEMPs, ocular vestibular-evoked myogenic potentials; HIT, head impulse test; ASNR, acoustically evoked, short latency negative response; EVAR, earth vertical axis rotation; OVAR, off-vertical axis rotation; vHIT, video head impulse testing; VNG, videonystagmography; cDVA, clinical dynamic visual acuity; VOR, vestibulo-ocular reflex; VST, vestibulospinal test battery; SHIMP, Suppression head impulse test; SVV, Subjective visual vertical.

## Discussion

Hearing loss is the most common sensory disorder in newborns, affecting approximately 0.1–0.2% of infants who are born deaf or hard-of-hearing (10). This incidence surpasses that of other congenital disorders detected through newborn screening, such as 13 out of 100,000 for haemoglobinopathies, 10 out of 100,000 for phenylketonuria, and 25 out of 100,000 for congenital hypothyroidism (11). The American Speech-Language-Hearing Association reports that the percentage of newborns with permanent hearing loss at birth is about 3% and that the number increases to 6% by the time children get old. Among the myriad of factors contributing to neonatal hearing loss, genetic causes, environmental exposures, and congenital infections—particularly cytomegalovirus (CMV)—play significant roles. Unlike postlingual hearing loss, prelingual deafness delays the development of auditory neural pathways, hindering normal speech and language acquisition. Early identification and timely intervention are crucial for preventing delays in language development. Treatment options include early intervention services, hearing aid amplification, and cochlear implantation. In addition to hearing loss, there is growing interest in understanding the impact of vestibular dysfunction on children with congenital hearing impairment. The vestibular system, located in the inner ears alongside the hearing organs, plays a vital role in maintaining balance, given the interconnectedness of the auditory and balance systems, disruptions in one system can often correlate with disruptions in the other (8).

It has been demonstrated that spatial hearing and balance are connected, in that a single fixed sound source can provide sufficient spatial cues for the central nervous system to better control postural stability (12). The vestibular system enables gaze stability during head movements through the vestibulo-ocular reflex (VOR) originating from the semicircular canals. It also participates in the perception of verticality and is involved in producing muscular synergies necessary for postural control and orientation via vestibulospinal reflexes. Thus, the vestibular system plays a crucial role in postural orientation and elaborates various postural adjustments essential for acquiring the different stages of postural−motor development (13). Almost 70% of children presenting with sensorineural hearing loss (SNHL) have vestibular system disturbances, with 20–40% having severe bilateral vestibular loss. Numerous causes of hearing loss can be linked to vestibular impairment. These include conditions such as complete partition (types 1–3), enlarged vestibular aqueduct syndrome, cochlear nerve deficiency, congenital CMV infection, meningitis, and exposure to ototoxic medications. In affected children, vestibular impairment May manifest progressively, either in conjunction with a similar Decline in hearing levels or independently of it. This population must be rehabilitated early in order to avoid motor and cognitional delayed development (14). However, research on the effects of vestibular dysfunction in this population remains limited.

This article reviews the current literature on the work-up of vestibular disorders with objective vestibular tests for diagnosing balance disorders in patients affected by congenital sensorineural hearing loss. Early diagnosis and subsequent rehabilitation plays a pivotal role in optimizing outcomes for patients with congenital hearing loss and vestibular disorders. By identifying and addressing these conditions promptly, we empower children to thrive and communicate effectively.

In our review, we found significant heterogeneity among the cases presented, both in terms of the etiology of hearing loss and regarding the vestibular tests used, the variability can be attributed to the fact that the various articles were written in different years and countries, thus reflecting diverse technological advancements and availability of materials. The wide time span during which the articles analyzed in this review were published (1960–2023) has led to a significant change in the use of vestibular tests for patient evaluation. In fact, overall, the most commonly used test was the caloric test, at least until 1990, when VEMPs (Vestibular Evoked Myogenic Potentials) were introduced and became widespread. Therefore, there is an inherent inconsistency in the types of tests used for the evaluation of vestibular disorders.

We excluded patients with cochlear implants (IC) from the review to concentrate on individuals who had not undergone surgical procedures that might impact the neurological organization of the inner ear. Several studies have demonstrated that children with cochlear implants exhibit altered sacculi, characterized by the absence of vestibular-evoked myogenic potentials (VEMPs) in response to click stimuli (15). Vestibular dysfunction can be found in almost 60% of post-IC patients (16).

Moreover, the results vary greatly because we decided to include both patients with non-syndromic hearing loss and those with more severe syndromes that also cause extreme anatomical alterations of the inner ear structures. This variation is reflected in the results, ranging from patients who are essentially asymptomatic without any motor balance disturbance to others experiencing symptoms such as vertigo attacks, nystagmus, postural instability, and complete motor disability. In some cases, despite evidence of vestibular test abnormalities, patients did not exhibit balance disturbances, indicating the remarkable adaptability of physiological systems (17). Additionally, including both unilateral and bilateral congenital deafness makes the results even more heterogeneous. Vestibular tests, in fact, have different interpretations depending on the case, as with the caloric test, which is not always appropriate in cases of bilateral congenital deafness. The wide age range of the patients included in the study is also a factor that contributes to the heterogeneity of the results. The outcomes of the tests can indeed be influenced by the patient’s cooperation, which clearly varies based on age. Hearing evaluation has been conducted using age appropriate tools. Hearing tests included pure tone audiometry, vocal audiometry, tympanometry, auditory brainstem response (ABR), and otoacoustic emissions. In addition to audiological and vestibular tests, patients underwent computed tomography (CT) and magnetic resonance imaging (MRI) to identify congenital anomalies of the vestibular apparatus such as enlarged vestibular aqueduct syndrome (EVA) or CHARGE syndrome.

Genetic tests were also conducted to assess potential genetic mutations in syndromes. Perinatal tests were performed to screen for CMV or other pathogens infection such as urine/blood PCR, maternal seroconversion, amniotic fluid sampling, and ophthalmologic evaluations were conducted in cases where alterations of vision were suspected (18). In the analyzed articles, assessments addressed the labyrinthine (semicircular canal) function, otolith function and integrated balance.

We found that the majority of studies after 1990 included for vestibular testing used VEMPS, cVEMPs or oVEMPS. The greatest advantage of the cVEMP and oVEMP tests is their ability to measure a different part of the vestibular system (i.e., the otolithic end organs), whereas videonystagmography (VNG) is typically used for performing caloric and rotational tests that assess the function of the horizontal semicircular canal and its connections with the superior vestibular nerve. VEMP examinations evaluate the right and left labyrinths separately, making VEMPs further useful in localizing the side of the lesion. Another advantage is that both of these tests are relatively quick and well-tolerated by patients.

Other tests utilized were rotatory test, caloric test with or without ENG, posturography, acoustically evoked, short latency negative response (ASNR), Dynamic visual acuity (DVA). Some authors have also tested dynamic balance in simulated conditions, using a virtual reality simulator.

Together with these tests, some authors have chosen to further investigate the balance of the patients using the Bruininks-Oseretsky Test of Motor proficiency-2 (BOT-2) and the challenging environmental assessment lab (CEAL) (19). The BOT-2 is an assessment tool for motor skills used to evaluate an individual’s ability to perform basic motor tasks, so it does not rely on objective response from specific gear but rely on the competence of qualified professionals. The BOT-2 consists of eight subtests that assess eye-hand coordination, manual dexterity, movement speed, static and dynamic balance, and muscle strength. The subtests are divided into two main areas: fine motor and gross motor skills.

Gerdsen et al. (20) have recently proposed a clinical vestibular testing algorithm in children to detect vestibular hypofunction. The first test which is used is vHIT since this test is quick, has low burden and high specificity, followed by caloric test o cVEMP depending on the age of the patient. In HIT, patients focus on a stationary target while experiencing sudden, small head rotations in the plane of each semicircular canal. This test evaluates the effectiveness of compensatory eye movements, assessing semicircular canal function studying the vestibulo-ocular reflex (VOR). vHIT test is valuable because it evaluates the function of both the horizontal and vertical semicircular canals, providing a comprehensive assessment of the vestibular system. By measuring the eye movements in response to rapid, small head impulses, the vHIT detects deficits in VOR, which is critical for maintaining stable vision during head movements. Combining vHIT with cervical and cVEMP and oVEMP tests enables a complete evaluation of all vestibular receptors, including both the semicircular canals (horizontal and vertical) and the otolithic organs (saccule and utricle). This integrated approach enhances the diagnostic accuracy for a wide range of vestibular disorders by assessing the entire vestibular apparatus.

This review highlights that the available data are limited due to significant heterogeneity, and there is no exact consensus on the methodology for conducting vestibular studies. This is allegedly mostly due to the deverseness of the conditions causing the hearing loss, the age of the patients and advance and availability of the technologies. In our study articles have been ruled out due to the absence of objective reproducible vestibular tests, so in the future we hope we’ll find a useful diagnostic algorithm in order to get early diagnosis for vestibular impairment in congenital deaf patients. In future studies, it will be crucial to ensure comprehensive reporting of all vestibular test results and to provide detailed specifications regarding which patients participated in specific tests within case series.

## Conclusion

Congenital hearing loss significantly impacts a child’s development, particularly language acquisition and communication skills. Early identification of hearing impairment allows for timely intervention, which can mitigate the adverse effects on language development. Individualized therapies, including hearing aids and cochlear implants, are most effective when initiated early.

In light of the frequent association between unilateral or bilateral congenital hearing loss and vestibular disorders, it is crucial to develop a diagnostic algorithm for the early detection of any associated vestibular issues. To date, there is still no consensus on which vestibular tests should be the first choice, largely due to the significant heterogeneity of studies in the literature and the diversity of patients. In the future, studies are needed to determine which vestibular test is most appropriate for assessing vestibular symptoms in patients with congenital deafness, taking into account the patient’s age and level of cooperation.

## Data Availability

The original contributions presented in the study are included in the article/supplementary material, further inquiries can be directed to the corresponding author.
